# Detection of Enteric Viruses in Children under Five Years of Age before and after Rotavirus Vaccine Introduction in Manhiça District, Southern Mozambique, 2008–2019

**DOI:** 10.3390/v16071159

**Published:** 2024-07-18

**Authors:** Percina Chirinda, Filomena Manjate, Marcelino Garrine, Augusto Messa, Nélio Nobela, Delfino Vubil, Tacilta Nhampossa, Sozinho Acácio, Quique Bassat, Karen L. Kotloff, Myron M. Levine, James P. Nataro, Jacqueline E. Tate, Umesh Parashar, Jason M. Mwenda, Pedro L. Alonso, Eva D. João, Inácio Mandomando

**Affiliations:** 1 Centro de Investigação em Saúde de Manhiça (CISM), Maputo 1929, Mozambique; percina.chirinda@manhica.net (P.C.); filomena.manjate@manhica.net (F.M.); marcelino.garrine@manhica.net (M.G.); augusto.junior@manhica.net (A.M.J.); nelio.nobela@manhica.net (N.N.); delfino.vubil@manhica.net (D.V.); tacilta.nhampossa@manhica.net (T.N.); sozinho.acacio@manhica.net (S.A.); quique.bassat@isglobal.org (Q.B.); alonso@ub.edu (P.L.A.); evajoao29@gmail.com (E.D.J.); 2 Global Health and Tropical Medicine, GHTM, Associate Laboratory in Translation and Innovation Towards Global Health, LA-REAL, Instituto de Higiene e Medicina Tropical, IHMT, Universidade NOVA de Lisboa, UNL, Rua da Junqueira 100, 1349-008 Lisbon, Portugal; 3 Instituto Nacional de Saúde (INS), Marracuene 1120, Mozambique; 4 ISGlobal, Hospital Clínic, Universitat de Barcelona, 08036 Barcelona, Spain; 5 Institució Catalana de Recerca I Estudis Avançats (ICREA), 08010 Barcelona, Spain; 6 Pediatrics Department, Hospital Sant Joan de Déu, Universitat de Barcelona, Esplugues, 08950 Barcelona, Spain; 7 CIBER de Epidemiología y Salud Pública, Instituto de Salud Carlos III, 28029 Madrid, Spain; 8 Center for Vaccine Development, School of Medicine, University of Maryland, Baltimore, MD 21201, USA; kkotloff@medicine.umaryland.edu (K.L.K.); mlevine@som.umaryland.edu (M.M.L.); 9 Department of Pediatrics, University of Virginia School of Medicine, Charlottesville, VA 22903, USA; jpn2r@virginia.edu; 10 Centers for Disease Control and Prevention (CDC), Atlanta, GA 30333, USA; jqt8@cdc.gov (J.E.T.); uap2@cdc.gov (U.P.); 11 World Health Organization (WHO), Regional Office for Africa, Brazzaville P.O. Box 2465, Congo; mwendaj@who.int; 12 Faculty of Medicine & Hospital Clínic, Universitat de Barcelona, 08036 Barcelona, Spain

**Keywords:** enteric viruses, diarrhoea, rotavirus vaccine, children, Mozambique

## Abstract

Enteric viruses are the leading cause of diarrhoea in children <5 years. Despite existing studies describing rotavirus diarrhoea in Mozambique, data on other enteric viruses remains scarce, especially after rotavirus vaccine introduction. We explored the prevalence of norovirus GI and GII, adenovirus 40/41, astrovirus, and sapovirus in children <5 years with moderate-to-severe (MSD), less severe (LSD) diarrhoea and community healthy controls, before (2008–2012) and after (2016–2019) rotavirus vaccine introduction in Manhiça District, Mozambique. The viruses were detected using ELISA and conventional reverse transcription PCR from stool samples. Overall, all of the viruses except norovirus GI were significantly more detected after rotavirus vaccine introduction compared to the period before vaccine introduction: norovirus GII in MSD (13/195, 6.7% vs. 24/886, 2.7%, respectively; *p* = 0.006) and LSD (25/268, 9.3% vs. 9/430, 2.1%, *p* < 0.001); adenovirus 40/41 in MSD (7.2% vs. 1.8%, *p* < 0.001); astrovirus in LSD (7.5% vs. 2.6%, *p* = 0.002); and sapovirus in MSD (7.1% vs. 1.4%, *p* = 0.047) and controls (21/475, 4.4% vs. 51/2380, 2.1%, *p* = 0.004). Norovirus GII, adenovirus 40/41, astrovirus, and sapovirus detection increased in MSD and LSD cases after rotavirus vaccine introduction, supporting the need for continued molecular surveillance for the implementation of appropriate control and prevention measures.

## 1. Introduction

Diarrhoea is the third leading global cause of morbidity and mortality in children under the age of five [1], causing an estimated 370.000 deaths worldwide in 2019 [2]. Enteric viruses are estimated to cause up to 75% of infectious diarrhoea cases, with rotavirus group A, norovirus, astrovirus, sapovirus, and enteric adenovirus being the main associated pathogens [3]. Noroviruses and sapoviruses are single-stranded RNA viruses and members of the *Caliciviridae* family. The *Norovirus* genus is classified into 10 genogroups (GI-GX), with only GI-GII and GIV being associated with human infections [4]. The *Sapovirus* genus is classified into 19 genogroups (GI-GXIX), with genogroups GI-GII and GIV-GV causing human infections [5]. Human astroviruses are also single-stranded RNA viruses of the family *Astroviridae*, genus *Mamastovirus*, with four species (MAstV-1, MAstV-6, MAstV-8, and MAstV-9) identified in humans, where MAstV-1 includes the human pathogenic genotypes (HAstV 1–8) [6]. Conversely, human adenoviruses are double-stranded DNA viruses and members of the family *Adenoviridae*, genus *Mastadenovirus*, classified into seven species (A-G) and fifty-two serotypes, with species F and serotypes 40 and 41 (adenovirus 40/41) associated with childhood diarrhoea [7,8].

Data on the burden of enteric viruses before the rotavirus vaccine introduction in Mozambique derive from the Global Enteric Multicenter Study (GEMS). This study aimed to determine the burden and aetiology of diarrhoeal disease in children under five years in developing countries across Africa (Kenya, Mali, Mozambique, The Gambia) and Asia (Bangladesh, India, Pakistan) between 2007 and 2012 [9]. GEMS reported rotavirus as the leading pathogen responsible for an attributable fraction of 35% of moderate-to-severe diarrhoea (MSD) cases and 20% for less severe diarrhoea (LSD) cases in infants in Mozambique [10,11]. Additionally, adenovirus 40/41 was ranked as the second viral aetiology with 2% of MSD-associated cases in the same age group [9]. These data supported the decision-making to introduce the Rotarix^®^ vaccine (GlaxoSmithKline Biologicals, Rixensart, Belgium) into the expanded programme of immunisation (EPI) of Mozambique in September 2015 [12], following the WHO recommendation to introduce the rotavirus vaccine in countries with a high disease burden [13]. After rotavirus vaccine introduction, the Centro de Investigação em Saúde de Manhiça (CISM) continued to monitor the trend and aetiology of diarrhoea in the Manhiça District to assess the vaccine’s impact and effectiveness.

Many countries have reported a significant reduction in rotavirus-associated diarrhoea after rotavirus vaccine introduction, along with an increase in the prevalence of other enteric viruses, such as norovirus and adenovirus 40/41 [14,15]. We have reported a decline in the prevalence of acute gastroenteritis and rotavirus positivity in infants with MSD in Manhiça District after the rotavirus vaccine introduction [16]; however, data reporting the circulation pattern of other enteric viruses are still scarce. Therefore, this study aims to explore and evaluate the contribution of norovirus GI and GII, sapovirus, astrovirus, and adenovirus 40/41 among MSD and LSD cases and community healthy controls under five years of age before and after rotavirus vaccine introduction in Manhiça District, Southern Mozambique.

## 2. Materials and Methods

### 2.1. Site Description

The study was conducted in the Manhiça District, a rural area located 80 km north of Maputo City (capital) in Southern Mozambique. Manhiça has a sub-tropical climate, characterised by a warm and rainy season from November to April and a cool and dry season during the rest of the year. The geographical and socio-demographic characteristics of the Manhiça District community were already described elsewhere [17,18]. Before rotavirus vaccine introduction, diarrhoea cases were enrolled at six health facilities: Manhiça District Hospital, Ilha Josina, Maragra, Malavele, Nwamatibjana, and Taninga health centres. After rotavirus vaccine introduction, cases were enrolled at three health facilities (Manhiça District Hospital, Xinavane Rural Hospital, and Maragra Health Centre). During both study periods, controls were enrolled from the Manhiça District community.

### 2.2. Study Design

We performed a sub-analysis of two case–control studies: the GEMS (before rotavirus vaccine introduction: 2007–2012) and the diarrheal diseases surveillance platform (after rotavirus vaccine introduction: 2015–2019). The study design, methodology, and inclusion criteria of both studies were similar and have been previously described [9,19]. Briefly, diarrhoea cases aged 0–59 months were enrolled in either the MSD or LSD groups. Diarrhoea was defined as the occurrence of three or more loose, liquid, or watery stools within 24 h [9]. MSD cases were those presenting with diarrhoea requiring hospitalisation and intravenous rehydration, while LSD cases comprised children with diarrhoea seeking care at outpatient visits without criteria for hospitalisation [9]. Controls were healthy children without diarrhoea from the community, matched with the index case (LSD and MSD) by age, sex, and neighbourhood. The participants were stratified into three age groups: 0–11, 12–23, and 24–59 months. In the GEMS, data from MSD cases and their respective controls were collected from December 2007 to November 2012. While, LSD cases and their controls were included in the last year of the study (November 2011–November 2012). There were no surveillance activities from 2013 to 2014. The diarrhoeal diseases surveillance platform collected MSD case data from September 2015 to December 2019, coinciding with the rotavirus vaccine introduction. Furthermore, LSD cases and controls (for MSD and LSD) data collection were included from April 2017 to December 2019.

### 2.3. Sample Collection

Stool samples were collected using a polyethene container and refrigerated in a cool box with a cooler block (2–8 °C) until delivery to CISM’s laboratories, where sample aliquots were frozen at −80 °C without preservatives until processing.

### 2.4. Laboratory Testing

#### 2.4.1. Enzyme-Linked Immunosorbent Assay (ELISA) for Virus Detection

Adenovirus was detected using the commercial ELISA kit ProSpecT Adenovirus Microplate (Prospect^®^ Adenovirus, Oxoid, Ltd., Hampshire, UK). Positive samples from the initial adenovirus ELISA were further tested for enteric adenovirus serotypes 40/41 using the ELISA kit Premier Adenoclone (Meridian Bioscience, Cincinnati, OH, USA).

#### 2.4.2. Multiplex Reverse Transcription Polymerase Chain Reaction (RT-PR) for Virus Detection

Viral RNA was extracted from stool supernatant using the QIAamp Viral RNA mini kit (QIAGEN, Hilden, Germany) according to the manufacturer’s protocol and screened by RT-PCR for detection of norovirus GI and GII, astrovirus, and sapovirus as previously described [20]. Briefly, RNA was synthesised to cDNA using random primers and an RT system (SuperScript III^®^ Reverse Transcriptase, Invitrogen, Waltham, MA, USA). After cDNA synthesis, multiplex PCR was conducted using specific primers (Table S1), and PCR products were electrophoresed on a 1.5% agarose gel, stained with 0.5 µg/mL ethidium bromide, and visualised under ultraviolet light in a trans-illuminator imaging gel documentation system (Bio-Rad Laboratories, Hercules, CA, USA).

### 2.5. Ethical Approval

Both GEMS and the diarrheal diseases platform study protocols were approved by the National Bioethics Committee for Health of Mozambique, CNBS (IRB 00002657), under the references 11/CNBS/07 and 209/CNBS/15, respectively.

### 2.6. Data Management and Statistical Analysis

A master database combining the data from the two studies (GEMS and the diarrheal diseases surveillance platform) was created, including clinical, epidemiological, and laboratory information. The comparison of the viruses’ frequencies before and after rotavirus vaccine introduction periods was performed separately for MSD, LSD cases and controls. To compare the seasonality trends between the two study periods, we considered the rainy season as the period from November to April and the dry season from May to October. All the data analyses were performed using STATA version 14.1 (StataCorp LP, College Station, TX, USA), and Chi-square or Fisher’s exact tests were used for the comparison of categorical variables, as appropriate. We considered a significance level of 5%.

## 3. Results

### 3.1. Characteristics of the Study Population

Overall, 4634 stool samples from children under five years of age were available for analysis, among which 1779 (38.4%) were from cases and 2855 (61.6%) from controls. Around 60.8% (1081/1779) of cases were MSD (886 before rotavirus vaccine introduction and 195 after vaccine introduction), and 39.2% (698/1779) were LSD (430 before vaccine introduction and 268 after vaccine introduction). Controls comprised 2380 (83.4%) samples collected before vaccine introduction and 475 (16.6%) collected after vaccine introduction. The characteristics of the study population are shown in Table 1.

### 3.2. Frequency of Enteric Viruses among MSD and LSD Cases and Controls before and after Rotavirus Vaccine Introduction

Overall, from the 4643 participants, norovirus GII (3.0%, n = 137) and sapovirus (2.8%, n = 129]) were the most detected viruses, followed by astrovirus (1.9%, n = 88), adenovirus 40/41 (1.8%, n = 84), and norovirus GI (1.4%, n = 65).

**Figure 1:**
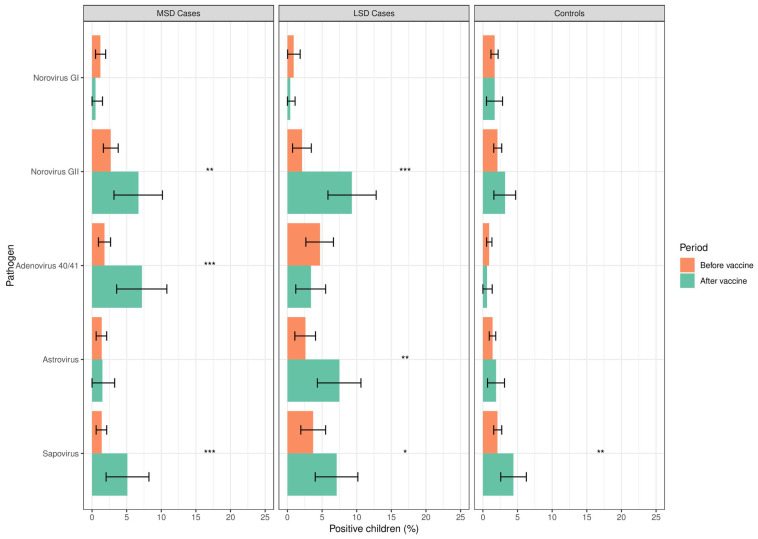
Frequency of enteric viruses among children <5 years of age with MSD, LSD, and controls before (2008–2012) and after (2016–2019) rotavirus vaccine introduction in Manhiça District, Mozambique. MSD: moderate-to-severe diarrhoea; LSD: less severe diarrhoea; controls: healthy children (without diarrhoea) from the community. * *p* ≤ 0.05; ** *p* ≤ 0.01; *** *p* ≤ 0.001. The error bars indicate 95% confidence intervals.

We observed a significant increase in the frequency of norovirus GII in MSD cases after vaccine introduction compared to the period before vaccine introduction (13/195, 6.7% vs. (24/886, 2.7%, respectively; *p* = 0.006). The same pattern was observed for adenovirus 40/41 (14/195, 7.2% vs. 16/886, 1.8%; *p* < 0.001) and sapovirus (10/195, 5.1% vs. 12/886, 1.4%; *p* = 0.001). Similarly, among LSD cases, there was a significant increase in the frequencies of norovirus GII (25/268, 9.3% vs. 9/430, 2.1%; *p* < 0.001), astrovirus (20/268, 7.5% vs. 11/430, 2.6%; *p* = 0.002) and sapovirus (19/268, 7.1% vs. 16/430, 3.7%; *p* = 0.047) after vaccine introduction. In contrast, sapovirus was the only virus among controls with a significant increase (51/2380, 2.1% vs. 21/475, 4.4%; *p* = 0.004) after vaccine introduction (Figure 1).

### 3.3. Frequency of Enteric Viruses among Cases and Controls According to the Age Strata before and after Rotavirus Vaccine Introduction

**Figure 2:**
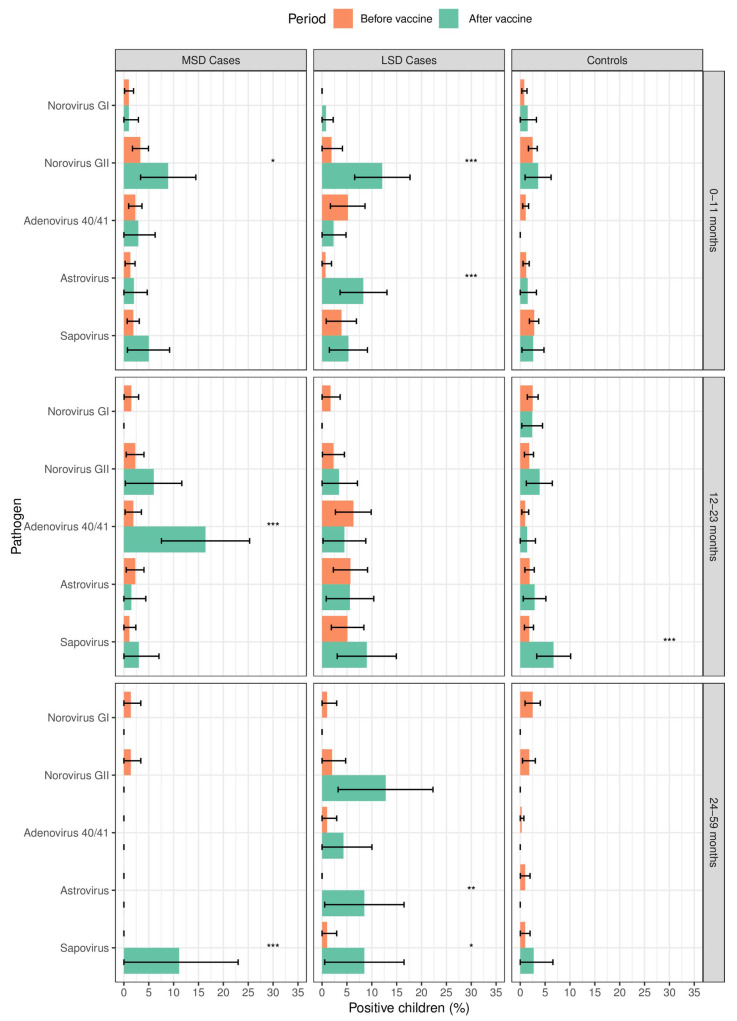
Frequency of enteric viruses among LSD and MSD cases and controls before (2008–2012) and after (2016–2019) rotavirus vaccine introduction, according to the age strata in Manhiça District, Mozambique. MSD: moderate-to-severe diarrhoea; LSD: less severe diarrhoea; controls: children without diarrhoea from the community. * *p* ≤ 0.05; ** *p* ≤ 0.01; *** *p* ≤ 0.001. The error bars indicate the 95% confidence intervals.

Norovirus GII significantly increased after vaccine introduction among MSD (9/101, 8.9% vs. 16/480, 3.3%; *p* = 0.012) and LSD cases (16/132, 12.1% vs. 3/155, 1.9%; *p* = 0.001) aged 0–11 months, followed by astrovirus among LSD cases (11/132, 8.3% vs. 1/155, 0.7%; *p* = 0.001) compared to the period before rotavirus vaccine introduction. In addition, among the 12–23 months age strata, we observed an increase in adenovirus 40/41 in MSD (11/67, 16.4% vs. 5/266, 1.9%; *p* < 0.001) and sapovirus in controls (14/208, 6.7% vs. 14/797, 1.8%; *p* < 0.001). Children aged 24–59 months had increased frequencies of norovirus GII in LSD (6/47, 12.8% vs. 2/100, 2%; *p* = 0.007), astrovirus in LSD (4/47, 8.5% vs. 0/100, 0%; *p* = 0.001) and sapovirus in MSD (3/27, 11.1% vs. 0/140, 0%; *p* < 0.001) and LSD (4/47, 8.5% vs. 1/100, 1%; *p* = 0.019) after rotavirus vaccine introduction (Figure 2).

### 3.4. Seasonality of Enteric Viruses before and after Rotavirus Vaccine Introduction

Astrovirus was mostly detected in cases in the dry season than in the rainy season after rotavirus vaccine introduction (16/214, 7.0% vs. 7/249, 3.0%, *p* = 0.021), while no specific pattern was observed before vaccine introduction (7/553, 1.0% vs. 16/763, 2.0%, *p* = 0.256). In contrast, adenovirus 40/41 (in cases [27/763, 4.0% vs. 5/553, 2.0%, *p* = 0.036] and in controls [17/1337, 1.0% vs. 5/1044, 0.5%, *p* = 0.045]) and norovirus GI (in controls [29/1337, 2.0% vs. 11/1044, 1.0%, *p* = 0.035]) were mostly detected in the rainy season before rotavirus vaccine introduction and did not show any specific pattern after vaccine introduction (Table S2). The seasonal distribution of the enteric viruses among cases and controls is presented in Table S2, and the monthly detection rates of the enteric viruses by year among cases and controls are presented in Figure S1 and Figure S2, respectively.

## 4. Discussion

We report the detection of enteric viruses in children with diarrhoea and healthy community controls before and after rotavirus vaccine introduction in Manhiça District, southern Mozambique. Despite the low overall frequencies, we documented a significant increase in norovirus GII (in MSD and LSD cases), adenovirus 40/41 (in MSD cases), astrovirus (in LSD cases), and sapovirus (in MSD and LSD cases, and controls) after rotavirus vaccine introduction. 

Norovirus GII was the predominant virus, which significantly increased after rotavirus vaccine introduction among MSD and LSD cases aged 0–11 months. These findings suggest the increasing importance of norovirus as a cause of diarrhoea in Mozambique, especially due to the decline of rotavirus-associated cases after vaccine introduction [16]. The same trend was observed in previous reports from Kenya, Brazil, Colombia, and Nicaragua after rotavirus vaccine introduction [21,22,23,24]. Furthermore, the increased frequencies in adenovirus 40/41 and sapovirus among MSD cases aged 12–23 months and 24–59 months suggest the important contribution of these viruses in the aetiology of severe diarrhoea. Some studies reported adenovirus 40/41 as one of the leading pathogens associated with diarrhoea in children after rotavirus vaccine introduction and sapovirus as the second leading pathogen after norovirus [22,25]; however, specificities of the study populations and differences in study designs may explain this feature. On the other hand, the rise in sapovirus positivity among controls aged 12–23 months could imply that the virus is present in the community, even if it is not causing diarrhoea, suggesting a link with previous infections [26]. The detection of astrovirus increased only in LSD cases after rotavirus vaccine introduction, and this finding is consistent with the recently published data from the Vaccine Impact on Diarrhoea in Africa (VIDA) study, which showed a strong association of astrovirus with MSD cases, although causing less severe infection [27]. Additionally, data from other studies characterised astrovirus diarrhoea as acute, mild, and self-limiting, being severe in immunocompromised patients [28,29,30,31]. 

Regarding the seasonality, the discrepancies observed between the two study periods for astrovirus, adenovirus 40/41, and norovirus GI may be due to the small number of positive cases detected throughout the study. Moreover, in agreement with previous reports, there is still divergence in showing a seasonality pattern for these viruses [14,15,32,33,34].

This study had some limitations. First, the lack of data from 2013 to 2014 may have affected the monitoring of the studied viruses in the last period before the vaccine introduction. Using ELISA for the detection of adenovirus 40/41 and conventional RT-PCR for the detection of the other viruses investigated in this study may have led to an underestimation of the frequency of adenovirus 40/41, as molecular methods have been proven to be more sensitive [35].

## 5. Conclusions

We observed a significant increase in norovirus GII, adenovirus 40/41, astrovirus, and sapovirus in diarrhoea cases after rotavirus vaccine introduction in Manhiça District, Mozambique. These findings support the need for continued molecular surveillance, as well as an expansion to other regions in the country for the design and implementation of appropriate control and prevention measures. 

## Figures and Tables

**Table 1:** Demographic characteristics of diarrhoea cases and community controls enrolled in the Manhiça District before (2008–2012) and after (2016–2019) rotavirus vaccine introduction.

	Cases N = 1779				Controls N = 2855	
	MSD N = 1081		LSD N = 698			
Characteristics	Before Vaccine [N = 886] n (%)	After Vaccine [N = 195] n (%)	Before Vaccine [N = 430] n (%)	After Vaccine [N = 268] n (%)	Before Vaccine [N = 2380] n (%)	After Vaccine [N = 475] n (%)
**Age strata**						
**0–11 months**	480 (54.2)	101 (51.8)	155 (30.0)	132 (49.3)	1184 (49.7)	195 (41.0)
**12–23 months**	266 (30.0)	67 (34.4)	175 (40.7)	89 (33.2)	797 (33.5)	208 (43.8)
**24–59 months**	140 (15.8)	27 (13.8)	100 (23.3)	47 (17.5)	399 (16.8)	72 (15.2)
**Sex**						
**Male**	527 (59.5)	115 (59.0)	236 (54.9)	147 (54.9)	1427 (60.0)	256 (53.9)
**Female**	359 (40.5)	80 (41.0)	194 (45.1)	121 (45.1)	953 (40.0)	219 (46.1)
**Rotavirus vaccination status**		N = 175		N = 254		N = 452
Vaccinated *	NA	137 (78)	NA	220 (87)	NA	364 (81)
**Unvaccinated ^#^**	NA	38 (22)	NA	34 (13)	NA	88 (19)

* Vaccinated children that received at least one dose of the vaccine (vaccine introduced in September 2015); # unvaccinated children eligible for vaccination, with no vaccine reception record; MSD: moderate-to-severe diarrhoea; LSD: less severe diarrhoea; controls: children without diarrhoea from the community; NA: not applicable (period before rotavirus vaccine introduction).

## Data Availability

All data are included in the manuscript.

## Supplementary Materials

The following supporting information can be downloaded at https://www.mdpi.com/article/10.3390/v16071159/s1, Table S1: List of primer sequences and amplicon sizes used in the RT-PCR multiplex to detect norovirus GI and GII, sapovirus, and astrovirus; Table S2: Seasonality of detection of enteric viruses in children under 5 years of age before (2008–2012) and after (2016–2019) rotavirus vaccine introduction in Manhiça District, Mozambique; Figure S1: Monthly detection rate of enteric viruses in the pre- and post-rotavirus vaccine introduction periods among diarrhoea cases before (2008–2012) and after (2016–2019) rotavirus vaccine introduction in Manhiça District, Mozambique; Figure S2: Monthly detection rate of enteric viruses among community controls before (2008–2012) and after (2016–2019) rotavirus vaccine introduction in Manhiça District, Mozambique.
