# Supplementary material for: Detection of Enteric Viruses in Children under Five Years of Age before and after Rotavirus Vaccine Introduction in Manhiça District, Southern Mozambique, 2008–2019
Source: Viruses. 2024 Jul 18;16(7):1159. doi: 10.3390/v16071159 (PMC11281453; doi:10.3390/v16071159)
Supplement: Supplementary file 1 [file viruses-16-01159-s001.zip › Supplementary Figure S1_JUNE2024.pdf]

Supplementary Figure S1

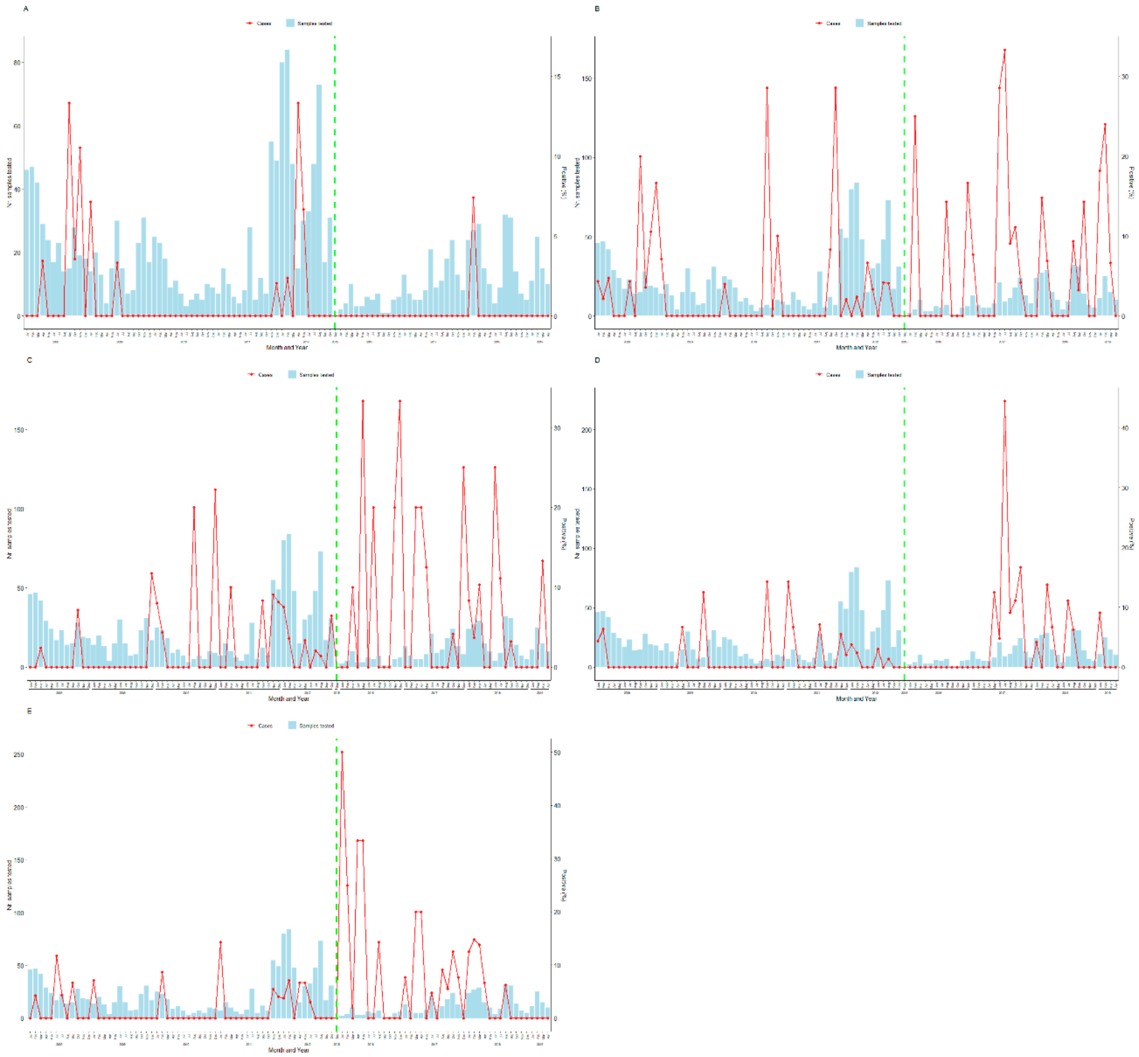

Figure S1. Monthly detection rate of enteric viruses in pre and post-rotavirus vaccine introduction periods among diarrhoea cases before (2008-2012) and after (2016-2019) rotavirus vaccine introduction in Manhiça District, Mozambique. (**A**) norovirus GI; (**B**) norovirus GII; (**C**) adenovirus 40/41; (**D**) astrovirus and (**E**) sapovirus. The Green dashed line indicates vaccine introduction time point in September 2015. There was no surveillance activity from 2013-2014.
