# Supplementary material for: Detection of Enteric Viruses in Children under Five Years of Age before and after Rotavirus Vaccine Introduction in Manhiça District, Southern Mozambique, 2008–2019
Source: Viruses. 2024 Jul 18;16(7):1159. doi: 10.3390/v16071159 (PMC11281453; doi:10.3390/v16071159)
Supplement: Supplementary file 1 [file viruses-16-01159-s001.zip › Supplementary Table S1_JUNE2024.pdf]

Supplementary Table S1

Table S1. List of Primer sequences and amplicon sizes used in the RT-PCR multiplex to detect norovirus GI and GII, sapovirus and astrovirus*

Virus	Primer	Primer sequence 5´-3´	Amplicon bp
Norovirus GI	G1SKR	CCAACCCARCCATTRTACA	330
	G1SKF	CTGCCCGAATTYGTAAATGA	
Norovirus GII	G2SKR	CCRCCNGCATRHCCRTTRTACAT	387
	COG2F	CARGARBCNATGTTYAGRTGGATGAG	
Sapovirus	SLV5749	CGGRCYTCAAAVSTACCBCCCCA	434
	SLV5317	CTCGCCACCTACRAWGCBTGGTT	
Astrovirus	82b	GTGAGCCACCAGCCATCCCT	719
	PreCAP1	GGACTGCAAAGCAGCTTCGTG	

*Reference: Panchalingam S, Antonio M, Hossain A, Mandomando I, Ochieng B, Oundo J, et al. Diagnostic Microbiologic Methods in the GEMS-1 Case/Control Study. Clinical Infectious Diseases 2012; 55:S294–302. https://doi.org/10.1093/cid/cis754.
