# Supplementary material for: Detection of Enteric Viruses in Children under Five Years of Age before and after Rotavirus Vaccine Introduction in Manhiça District, Southern Mozambique, 2008–2019
Source: Viruses. 2024 Jul 18;16(7):1159. doi: 10.3390/v16071159 (PMC11281453; doi:10.3390/v16071159)
Supplement: Supplementary file 1 [file viruses-16-01159-s001.zip › Supplementary Table S2_JUNE2024.pdf]

Supplementary Table S2

Table S2. Seasonality of detection of enteric viruses in children under 5 years of age before (2008-2012) and after (2016-2019) rotavirus vaccine introduction in Manhiça District, Mozambique.

Virus	Before vaccine			After vaccine		
	Rainy season n (%)	Dry season n (%)	p value	Rainy season n (%)	Dry season n (%)	p value
**Cases**	N=763	N=553		N=249	N=214	
Norovirus GI	9 (1.0)	6 (1.0)	0.873	2 (1.0)	0 (0)	0.502
Norovirus GII	17 (2.0)	16 (3.0)	0.446	18 (7.0)	20 (9.0)	0.408
Adenovirus 40/41	27 (4.0)	9 (2.0)	**0.036**	16 (4.0)	7 (3.0)	0.119
Astrovirus	16 (2.0)	7 (1.0)	0.256	7 (3.0)	16 (7.0)	**0.021**
Sapovirus	21 (3.0)	7 (1.0)	0.065	19 (8.0)	10 (5.0)	0.190
**Controls**	N=1,336	N=1,044		N=243	N=232	
Norovirus GI	29 (2.0)	11 (1.0)	**0.035**	5 (2.0)	3 (1.0)	0.517
Norovirus GII	30 (2.0)	21 (2.0)	0.696	7 (3.0)	8 (3.0)	0.724
Adenovirus 40/41	17 (1.0)	5 (0.5)	**0.045**	1 (0.4)	2 (1.0)	0.483
Astrovirus	21 (2.0)	12 (1.0)	0.382	2 (1.0)	7 (3.0)	0.080
Sapovirus	24 (2.0)	27 (3.0)	0.187	12 (5.0)	9 (4.0)	0.575

Cases: children presenting with moderate-to-severe diarrhoea (MSD) and less-severe diarrhoea (LSD; Controls: children without diarrhoea from the community; Rainy season: period from November to April; Dry season: period from May to October.
